# Genome-Wide Expression Profiling and Phenotypic Analysis of Downstream Targets Identify the Fox Transcription Factor Jumeau as a Master Regulator of Cardiac Progenitor Cell Division

**DOI:** 10.3390/ijms252312933

**Published:** 2024-12-01

**Authors:** M. Rezaul Hasan, Andrew J. Kump, Evelyn C. Stepaniak, Manoj Panta, Kuncha Shashidhar, Rajnandani Katariya, Mofazzal K. Sabbir, Kristopher R. Schwab, Mark H. Inlow, Ye Chen, Shaad M. Ahmad

**Affiliations:** 1 Department of Biology, Indiana State University, Terre Haute, IN 47809, USA; 2 The Center for Genomic Advocacy, Indiana State University, Terre Haute, IN 47809, USA; 3 Rich and Robin Porter Cancer Research Center, Indiana State University, Terre Haute, IN 47809, USA; 4 Indiana University School of Medicine, Indianapolis, IN 46202, USA; 5 Department of Mathematical Sciences, Indiana State University, Terre Haute, IN 47809, USA; 6 Department of Mathematics and Statistics, Northern Arizona University, Flagstaff, AZ 86001, USA

**Keywords:** Fox transcription factors, Forkhead box transcription factors, RNA-seq, ChIP-seq, genome-wide transcription expression profiling, transcriptional regulation, cardiac progenitor cell division, heart development and disease, cardiogenesis, *Drosophila*

## Abstract

Forkhead box (Fox) transcription factors (TFs) mediate multiple conserved cardiogenic processes in both mammals and *Drosophila*. Our prior work identified the roles of two *Drosophila* Fox genes, *jumeau* (*jumu*) and *Checkpoint suppressor 1-like* (*CHES-1-like*), in cardiac progenitor cell specification and division, and in the proper positioning of cardiac cell subtypes. Fox TF binding sites are also significantly enriched in the enhancers of genes expressed in the heart, suggesting that these genes may play a core regulatory role in one or more of these cardiogenic processes. We identified downstream targets of Jumu by comparing transcriptional expression profiles of flow cytometry-sorted mesodermal cells from wild-type embryos and embryos completely lacking the *jumu* gene and found that genes with functional annotation and ontological features suggesting roles in cell division were overrepresented among Jumu targets. Phenotypic analysis of a subset of these targets identified 21 *jumu*-regulated genes that mediate cardiac progenitor cell division, one of which, *Retinal Homeobox* (*Rx*), was characterized in more detail. Finally, the observation that many of these 21 genes and/or their orthologs exhibit genetic or physical interactions among themselves indicates that Jumu is a master regulator acting as a hub of a cardiac progenitor cell division-mediating network.

## 1. Introduction

Cardiogenesis involves the integration of multiple developmental processes regulated both spatially and temporally by a complex network of genes and signaling pathways. Pivotal to this regulatory network are the Forkhead box (Fox) transcription factors (TFs), proteins characterized by their conserved “forkhead” or “winged-helix” DNA-binding domain [1,2]: at least eight Fox TF-encoding genes (*Foxa2*, *Foxc1*, *Foxc2*, *Foxh1*, *Foxm1*, *Foxo1*, *Foxp1*, and *Foxp4*) are required for proper cardiac development in vertebrates [3,4,5,6,7,8,9,10,11,12,13,14,15,16,17,18], while mutations in four Fox genes (*FOXC1*, *FOXC2*, *FOXH1*, and *FOXP1*) are associated with human congenital heart defects [19,20,21,22,23,24,25,26]. Despite their obvious importance, however, relatively little is known about the downstream targets and molecular pathways utilized by these Fox TFs to bring about embryonic heart development [27].

Given both the amenability of *Drosophila melanogaster* to genetic analysis and the remarkable conservation of genes and molecular processes in heart development between mammals and *Drosophila* [28,29,30,31,32,33,34], we have been using the latter to study the cardiogenic roles of the Fox genes. Our prior work identified the conserved roles of two *Drosophila* Fox genes, *jumeau* (*jumu*) and *Checkpoint suppressor 1-like* (*CHES-1-like*) in specifying cardiac progenitor cells, and in bringing about their subsequent division into distinct cardiac cell subtypes. We showed that these two *Drosophila* Fox genes function redundantly to transcriptionally activate *heartless* (*htl*) and *frizzled* (*fz*). Since *htl* and *fz*, respectively, encode receptors of the FGF-signaling and Wnt-signaling pathways that are crucial for specifying cardiac progenitors, the absence of both Fox genes leads to incomplete heart specification [35]. We also demonstrated that both *jumu* and *CHES-1-like* mediate three distinct categories of cardiac progenitor cell divisions that determine the correct subtypes and numbers of cells constituting the heart—asymmetric cell divisions, symmetric cell divisions, and cell divisions at an earlier stage that produce the cardiac precursor cells—by regulating the activity of the conserved kinase Polo [36,37,38], a finding which suggested that similar conserved Fox TF-regulated cell division processes may also mediate cardiomyocyte proliferation in mammals [12,39,40]. Furthermore, we found that Fox TF binding sites were significantly overrepresented in known or putative enhancers of multiple genes expressed in the heart [36,41], suggesting that heart development may require additional Fox TF-regulated genes besides *htl*, *fz*, and *polo*. In turn, this raised questions as to what these additional Fox TF-regulated downstream genes might be and which cardiogenic processes they might mediate.

Here, in order to address those questions, we first identified genes regulated by one of the Fox TFs, Jumu, by comparing transcriptional expression profiles of mesodermal cells from wild-type and *jumu* loss-of-function *Drosophila* embryos. Analysis of functional annotation and gene ontology features revealed that genes putatively associated with cell division were disproportionately enriched among those that were transcriptionally activated by Jumu, suggesting a possible role for these *jumu*-activated genes in Fox TF-mediated cardiac progenitor cell division. We confirmed this hypothesis by performing phenotypic analyses of mutant alleles of 21 of these Jumu-activated genes and finding that they did indeed lead to cardiac progenitor cell division defects. As proof of principle, we also performed a more detailed characterization of one of these genes, *Retinal Homeobox* (*Rx*), to identify the specific categories of cardiac progenitor cell divisions this *jumu*-activated gene mediates and to demonstrate that it is actually utilized by *jumu* to bring about these cell divisions. Chromatin immunoprecipitation data were used to order the *jumu*-activated genes based on their likelihood of being directly regulated by Fox TF binding. Notably, most of these 21 target genes or their orthologs exhibit genetic or physical interactions among themselves, suggesting that they define a cardiac progenitor cell division network regulated by Jumu.

## 2. Results

### 2.1. Identification of Genes Regulated by Jumu

While *jumu* has been implicated in the development of multiple organs and systems throughout the life of the fruit fly *Drosophila* [42,43,44,45,46,47,48,49,50], the two known *jumu*-mediated conserved cardiogenic processes in *Drosophila*, cardiac progenitor specification and subsequent cell divisions, occur in a subset of the mesoderm during embryonic stages 11 to 12 [35,36,37,38]. Thus, in order to identify the *jumu*-regulated genes involved in heart development, it was necessary to obtain and compare genome-wide transcriptional expression profiles of mesodermal cells at these stages from wild-type and *jumu* loss-of-function *Drosophila* embryos.

Since *twist* (*twi*) expression is one of the earliest pan-mesodermal markers [51], we used *twi-GAL4* [52] to drive a bright UAS-dicistronic enhanced GFP (*UAS-2EGFP*) [53] to fluorescently mark mesodermal cells for sorting via flow cytometry. One version of our strategy was designed to ensure that only the cells that both lacked *jumu* function and were also mesodermal would express GFP and thus be purified. To achieve this goal, we recombined the *twi-Gal4* construct with a chromosome containing a *jumu* null deficiency, *jumu^Df(3R)Exel6157^* (also known as *Df(3R)Exel6157*) in one strain, and the *UAS-2EGFP* transgene with the same *jumu^Df(3R)Exel6157^* null deficiency in a second strain. When these two strains are crossed, GFP expression occurs exclusively in mesodermal cells lacking *jumu* function; neither wild-type mesoderm nor *jumu*-deficient nonmesodermal cells show GFP expression in the resulting embryos.

In contrast, to obtain wild-type mesodermal cells, we simply crossed a strain homozygous for both the *twi-GAL4* and the *UAS-2EGFP* transgenes to a wild-type strain; only wild-type mesodermal cells expressed GFP in the resulting embryos.

Embryos from these crosses were aged to stages 11–12 before being gently dissociated to obtain single-cell suspensions. While *jumu* is an essential gene, and its elimination invariably results in lethality, *jumu*-deficient embryos develop and survive to at least stage 16, allowing these procedures to be implemented. Fluorescence-activated cell sorting (FACS) was used to purify GFP-expressing cells from each of these suspensions. Total RNA was isolated from each population of GFP-expressing cells and used to obtain transcriptional expression profiles for both wild-type mesoderm and mesoderm lacking *jumu* function via RNA sequencing (RNA-seq) and subsequent data analysis.

Genes regulated at a transcription level by *jumu* were expected to exhibit significant fold changes in mRNA expression levels in embryos lacking *jumu* function compared to wild-type. Using rather stringent criteria (log_2_FoldChange > 1 or log_2_FoldChange < −1 and False Discovery Rate < 0.1), our data analysis identified 1271 genes that were dysregulated, with 693 being repressed (log_2_FoldChange > 1) by *jumu* in the mesoderm and 578 being activated (log_2_FoldChange < −1) (Supplementary Table S1). A more relaxed set of fold change criteria (log_2_FoldChange > 0.5 or log_2_FoldChange < −0.5 and False Discovery Rate < 0.1) identified 2496 *jumu*-regulated genes, with 1250 being repressed and 1246 being activated by the Fox TF (Supplementary Table S1).

### 2.2. Functional Annotation Enrichment Analysis Implicates Jumu-Activated Genes in Cell Division

Next, we attempted to determine the cardiogenic processes in which the *jumu*-regulated genes might be involved. If many of these downstream genes are involved in the same cardiogenic process, then we would expect to see an overrepresentation of functional annotation terms or ontological features associated with that particular process. The DAVID knowledgebase [54,55] compiles and integrates genes and their functional and sequence annotations from a variety of public genomic resources (NCBI, Uniprot, Ensembl, Gene Ontology, KEGG, Reactome, etc.). Since *jumu*-regulated genes mediating a particular cardiogenic process are likely to be regulated in a similar fashion (either activated or repressed) by the Fox TF, we utilized the *jumu*-activated and the *jumu*-repressed pools of genes (Supplementary Tables S2A and S2C, respectively) independently to query the DAVID knowledgebase for overrepresentation of functional annotation terms. In both cases, we performed functional annotation clustering with all genes of the *Drosophila melanogaster* genome as background. None of the enriched annotation clusters of *jumu*-repressed mesodermal genes suggested obvious cardiogenic processes with the possible exception of Annotation Clusters 22 and 55, which featured the terms basement membrane and extracellular matrix (Supplementary Table S2B). Extracellular matrix (ECM) proteins, particularly the specialized subset comprising the basement membrane (BM), and their regulators have previously been shown to be important for embryonic heart morphogenesis and development [56,57,58].

In contrast, we found that the most enriched annotation cluster (Annotation Cluster 1) of *jumu*-activated mesodermal genes featured the terms mitosis, cell division, and cell cycle, suggesting a potential role for these genes in *jumu*-mediated cardiac progenitor cell division (Supplementary Table S2D). This hypothesis was further supported by the terms featured in the highly enriched Annotation Clusters 2 and 7 (microtubule and motor proteins), Annotation Cluster 4 (centromere and spindle midzone), Annotation Cluster 5 (centrosome and centriole), Annotation Cluster 8 (mitotic chromosome condensation), and Annotation Cluster 11 (mitotic cytokinesis), all of which are associated with different aspects of cell division (Supplementary Table S2D).

### 2.3. Phenotypic Analysis of 21 Jumu-Activated Genes Demonstrate That They Are Essential for Mediating Cardiac Progenitor Cell Division

The overrepresentation of functional annotation terms associated with cell division for *jumu*-activated mesodermal genes hints at, but does not necessarily confirm, a major role for many of these *jumu*-activated genes in mediating cardiac progenitor cell division. While this is a likely possibility given that *jumu* mediates three categories of cardiac progenitor cell division, it is not the only one, since *jumu* has also been shown to regulate cell division in other tissues and cell types such as both hemocytes [46,59] and ganglion mother cells [43]. If, however, many of the *jumu*-activated genes identified by our transcription expression profiling do indeed mediate cardiac progenitor cell division, then we would expect the loss of function of these genes to exhibit all or a subset of the cardiac progenitor cell division defects detected in *jumu* mutants. We elected to test this hypothesis by disrupting the function of a subset of *jumu*-activated mesodermal genes. Consequently, we first assessed which of the 1246 *jumu*-activated genes identified in our expression profiling analysis (Section 2.1) possessed null or strongly hypomorphic mutant alleles that were readily available from the Bloomington Drosophila Stock Center. A total of 185 *jumu*-activated genes satisfied this criterion. From this pool of 185, 21 genes were selected at random for phenotypic analysis: *α-Tubulin at 67C* (*αTub67C*), *Adenomatous polyposis coli 2* (*Apc2*), *barren* (*barr*), *Bub1-related kinase* (*BubR1*), *Cyclin-dependent kinase 2* (*Cdk2*), *Centromeric protein-C* (*Cenp-C*), *centromere identifier* (*cid*), *CENP-meta* (*cmet*), *gluon* (*glu*), *Inner centromere protein* (*Incenp*), *Kinesin-like protein at 61F* (*Klp61F*), *meiotic from* via *Salaria 332* (*mei-S332*), *nebbish* (*neb*), *pavarotti* (*pav*), *pimples* (*pim*), *Retinal Homeobox* (*Rx*), *scraps* (*scra*), *Structural maintenance of chromosomes 2* (*SMC2*), *sticky* (*sti*), *three rows* (*thr*), and *tumbleweed* (*tum*) (Table 1).

**Figure 1:**
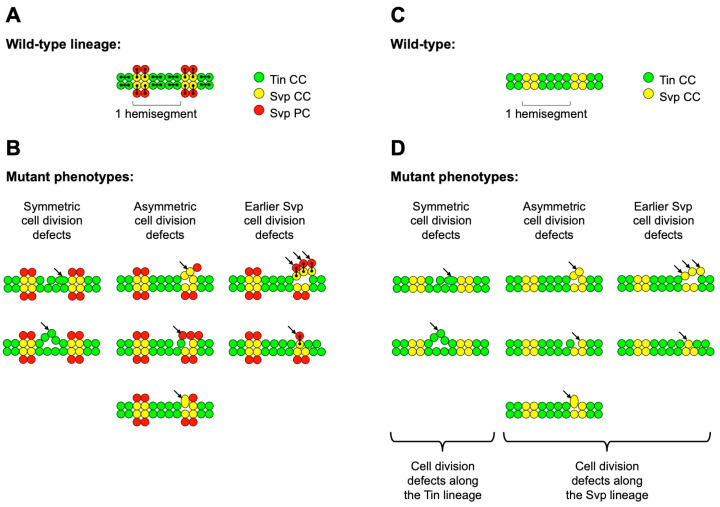
Schematic showing cell lineage relationships in a wild-type *Drosophila* embryonic heart and the expected phenotypes due to defects in cardiac progenitor cell division. (**A**) Lines connect daughter cells arising from the division of each progenitor cell in a wild-type heart. (**B**) Defects in symmetric cell division would result in an increase or reduction in the expected number of four Tin-CCs per hemisegment. Defective asymmetric cell division would result in an increase or reduction in the number of Svp-CCs accompanied by a corresponding decrease or increase in the number of Svp-PCs, or larger Svp-CC nuclei with missing corresponding Svp-PCs due to errors in karyokinesis. Errors at the earlier stage of cell division that produces the two Svp progenitors would result in hemisegments with either one or three pairs of Svp-CCs and Svp-PCs instead of the customary two pairs. (**C**) A wild-type heart where the Svp-PCs cannot be visualized. (**D**) The same set of symmetric, asymmetric, and earlier cardiac progenitor cell division defects that were illustrated in (**B**), in a context where the Svp-PCs cannot be visualized. Note that certain asymmetric cell division defects cannot be distinguished from earlier cardiac progenitor cell division defects when Svp-PCs cannot be detected. Cardiac progenitor cell division defects are indicated by arrows in (**B**,**D**).

The wild-type embryonic *Drosophila* heart consists of a metameric linear tube of 104 contractile cardial cells (CCs) surrounded by a sheath of pericardial cells (PCs) that perform supportive and nephrocytic roles [28]. The metameric nature of the heart ensures that every hemisegment from A2 to A7 exhibits the same repeated pattern of heart cells from anterior to posterior, two Seven up (Svp)-expressing cardial cells (Svp-CCs, yellow cells in Figure 1A) followed by four Tinman (Tin)-expressing cardial cells (Tin-CCs, green cells in Figure 1A); with the A8 cardiac hemisegments being truncated in having the two Svp-CCs followed by only two Tin-CCs. This is a consequence of a series of stereotypical and invariant series of cardiac progenitor cell divisions in the wild-type *Drosophila* embryo [28,60,61,62]. In each cardiac hemisegment, a cell division event at an earlier stage generates two Svp cardiac progenitor cells, with each Svp progenitor cell subsequently undergoing asymmetric cell division to produce an Svp-CC and an associated pericardial cell, an Svp-PC (Figure 1A, yellow and red cells, respectively). In contrast, two symmetric cell divisions give rise to four Tin-CCs (Figure 1A, green cells) per hemisegment from two Tin cardiac progenitor cells. Thus, collectively, these lineage relationships would allow us to use the numbers of Tin-CCs, Svp-CCs, and Svp-PCs in individual hemisegments of embryos mutant for a particular *jumu*-activated gene to determine which, if any, of these cardiac progenitor cell division categories the target gene is mediating. For example, if the gene was critical for asymmetric cell division, an increase or reduction in the number of Svp-CCs accompanied by a corresponding decrease or increase in the number of Svp-PCs would be detected in the mutant, or larger Svp-CC nuclei with missing corresponding Svp-PCs due to errors in karyokinesis would be observed (Figure 1B). Conversely, defects in symmetric cell division would manifest as deviations from the expected number of four Tin-CCs per hemisegment (Figure 1B). Finally, errors during the earlier stage of cell division which normally produce two Svp progenitor cells would result in hemisegments with either one or three Svp progenitors, giving rise ultimately to one or three pairs of Svp-CCs and Svp-PCs instead of the customary two pairs (Figure 1B).

**Figure 2:**
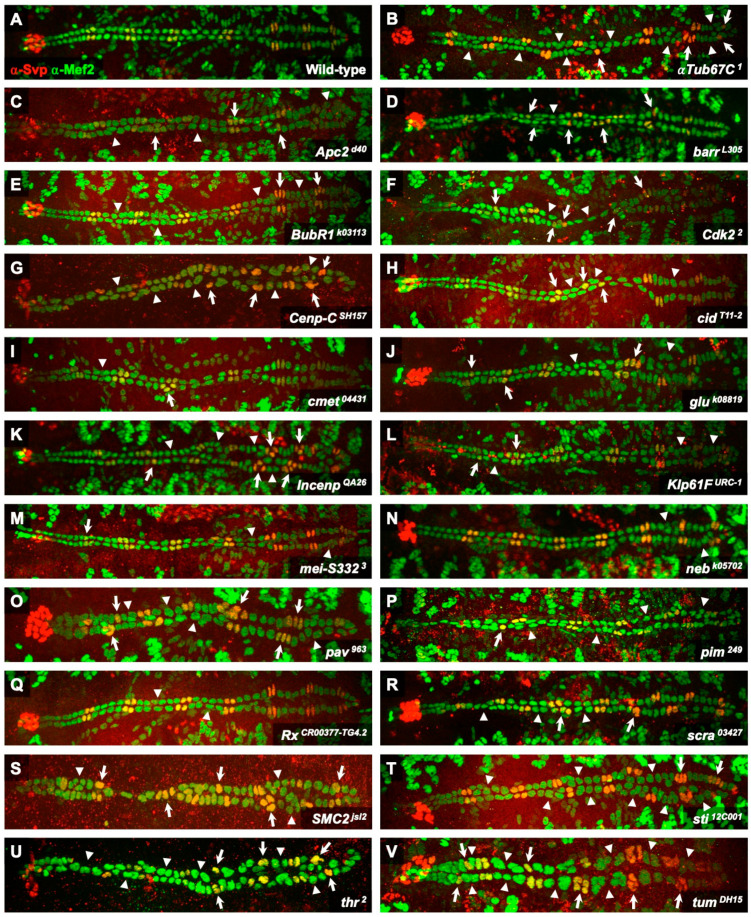
Cardiac progenitor cell division defects associated with mutations in 21 *jumu*-activated genes. (**A**) A heart from a wild-type embryo showing hemisegments consisting of two Svp-CCs (yellow) and four Tin-CCs (green). (**B**–**V**) Hearts from embryos homozygous for null or strongly hypomorphic mutations of the *jumu*-activated genes *αTub67C* (**B**), *Apc2* (**C**), *barr* (**D**), *BubR1* (**E**), *Cdk2* (**F**), *Cenp-C* (**G**), *cid* (**H**), *cmet* (**I**), *glu* (**J**), *Incenp* (**K**), *Klp61F* (**L**), *mei-S332* (**M**), *neb* (**N**), *pav* (**O**), *pim* (**P**), *Rx* (**Q**), *scra* (**R**), *SMC2* (**S**), *sti* (**T**), *thr* (**U**), and *tum* (**V**) exhibiting cardiac progenitor cell division defects along both the Svp lineage (arrows) that correspond to asymmetric or earlier cell division errors, and the Tin lineage (arrowheads) that correspond to symmetric cell division errors.

Introducing an *svp-lacZ* enhancer trap into every mutant and wild-type line and labeling them with antibodies for both Myocyte enhancer factor 2 (Mef2), which recognizes all CCs, and β-galactosidase, which marks both Svp-CCs and Svp-PCs, could have allowed us to distinguish and discriminate between Tin-CCs, Svp-CCs, and Svp-PCs, and determine which categories of cardiac progenitor cell division, if any, were mediated by each of these *jumu*-activated genes [36,37,38]. However, the introduction of this enhancer trap into all 21 mutant lines would have been overly time-consuming and inefficient, given that the primary question we wanted to address was whether one or more of these 21 genes mediated cardiac progenitor cell divisions at all. We therefore elected to label the embryos with antibodies specific to Mef2 and Svp (which marks the Svp-CCs exclusively) instead. This allowed us to distinguish the Tin-CCs (green) from Svp-CCs (yellow) and determine the numbers of each of these cell types in wild-type and appropriate mutant embryonic hearts ((Figure 1C,D and (Figure 2). Any deviation from the expected number of two Svp-CCs in a hemisegment would indicate a defect in cardiac progenitor cell division along the Svp lineage, i.e., in either asymmetric or earlier cell division, while a change in the expected number of Tin-CCs would indicate an error in symmetric cardiac progenitor cell division (Figure 1D).

**Figure 3:**
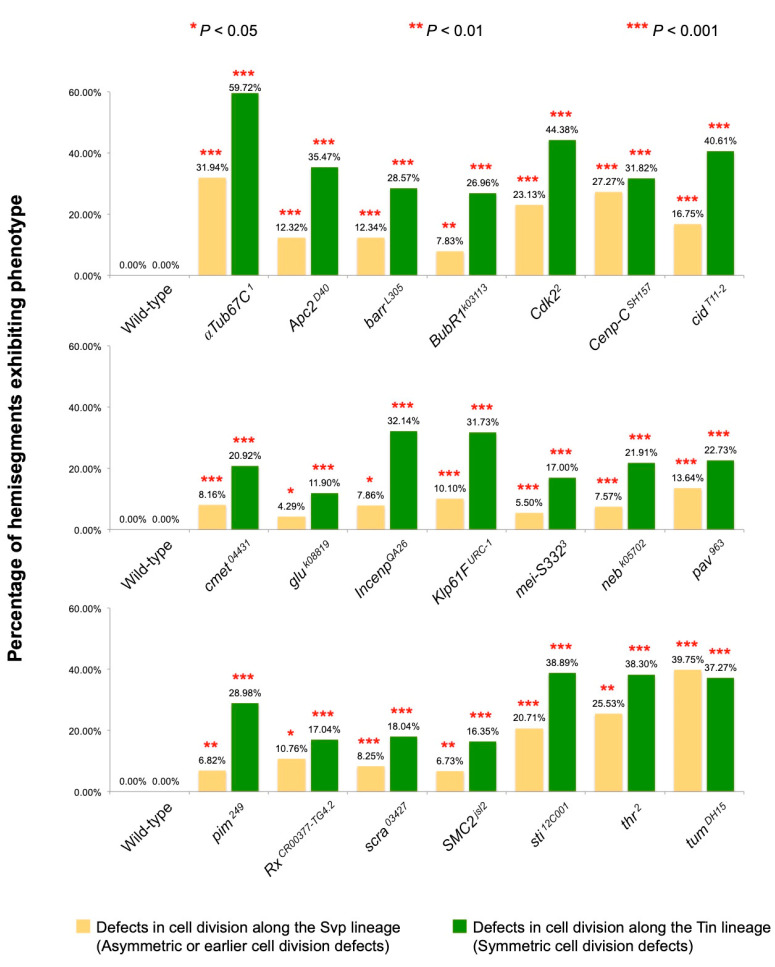
Percentage of hemisegments exhibiting cardiac progenitor cell division defects along the Svp lineage (yellow) and Tin lineage (green) for mutations in 21 *jumu*-activated genes. The number of embryos and hemisegments, respectively, examined for each genotype are as follows: wild-type = 14 and 196, *αTub67C* ^*1*^ = 12 and 144, *Apc2^d40^* = 16 and 203, *barr^L305^* = 12 and 154, *BubR ^k03113^* = 17 and 230, *Cdk2^2^*= 14 and 160, *Cenp-C* ^*SH157*^ = 14 and 176, *cid ^T11-2^* = 15 and 197, *cmet* ^*04431*^ = 15 and 196, *glu^k08819^* = 15 and 210, *Incenp^QA26^* = 10 and 140, *Klp61F^URC-1^* = 15 and 208, *mei-S332^3^* = 15 and 200, *neb^k05702^* = 18 and 251, *pav^963^* = 8 and 110, *pim^249^* = 14 and 176, *Rx ^CR00377-TG4.2^* = 17 and 223, *scra ^03427^* = 14 and 194, *SMC2 ^jsl2^* = 15 and 208, *sti^12C001^* = 15 and 198, *thr^2^* = 7 and 94, and *tum ^DH15^* = 14 and 161. The relative significance of each type of cell division defect in the mutants compared with wild-type is shown.

We used the criteria described above to examine embryos that were either wild-type or homozygous for null or strongly hypomorphic mutations of the 21 *jumu*-activated genes that we had selected. Of note, similar to the *jumu null* deficiency, all 21 of these mutant alleles are homozygous lethal, but the embryos develop and survive to at least stage 16, enabling us to perform these phenotypic analyses. Much to our surprise, our examination found that each and every one of these mutant genotypes exhibited a significant increase over wild-type in the fraction of hemisegments with fewer or excess Svp-CCs, corresponding to asymmetric cell division defects or earlier cell division defects affecting the number of Svp progenitors, and in the fraction of hemisegments showing deviations from the expected number of Tin-CCs, corresponding to symmetric cardiac progenitor cell division defects (Figure 2 and Figure 3; Supplementary Table S3). Collectively, by demonstrating that all 21 of these selected *jumu*-activated genes are necessary for bringing about proper cardiac progenitor cell divisions along both the Svp and Tin lineage, our results establish that a major role for *jumu*-activated downstream genes is indeed mediating cardiac progenitor cell divisions.

### 2.4. Expression Profiling of RNA from Flow Cytometry-Purified Mesodermal Cells Is More Sensitive at Identifying Cardiogenic Jumu-Regulated Genes than Whole Embryo RNA

In order to identify *jumu*-regulated genes potentially mediating cardiogenic processes, we compared transcription expression profiles from flow cytometry-purified mesodermal cells of the relevant genotypes instead of from entire wild-type and *jumu*-deficient embryos. We believed that the first option allowed us to eliminate or reduce the potentially confounding effects of *jumu* loss of function in non-cardiogenic processes in the rest of the embryo and thereby increased the sensitivity of our analysis. To test this premise, we examined whether the 21 *jumu*-activated genes mediating cardiac progenitor cell divisions that we initially identified via RNA-seq of flow cytometry-purified mesodermal cells would also have been recognized by reverse transcription quantitative real-time PCR (RT-qPCR) of RNA from wild-type and *jumu*-deficient whole embryos. Our RT-qPCR analysis of whole embryo RNA revealed that only 18 of these 21 genes showed significant (*p* ≤ 0.05) reduction in expression levels in *jumu* deficient embryos compared to wild-type (Supplementary Figure S1). The remaining three genes—*αTub67C*, *cid*, and *Incenp*—also exhibited reduced expression in embryos lacking *jumu*, but the reduction in expression levels obtained from whole embryos was not significant (*p* > 0.05). Collectively, our assessment indicates that our belief that using flow cytometry-purified mesodermal cells instead of whole embryos would yield higher sensitivity was not misplaced.

### 2.5. Rx Is Required for Three Distinct Categories of Cardiac Progenitor Cell Division

**Figure 4:**
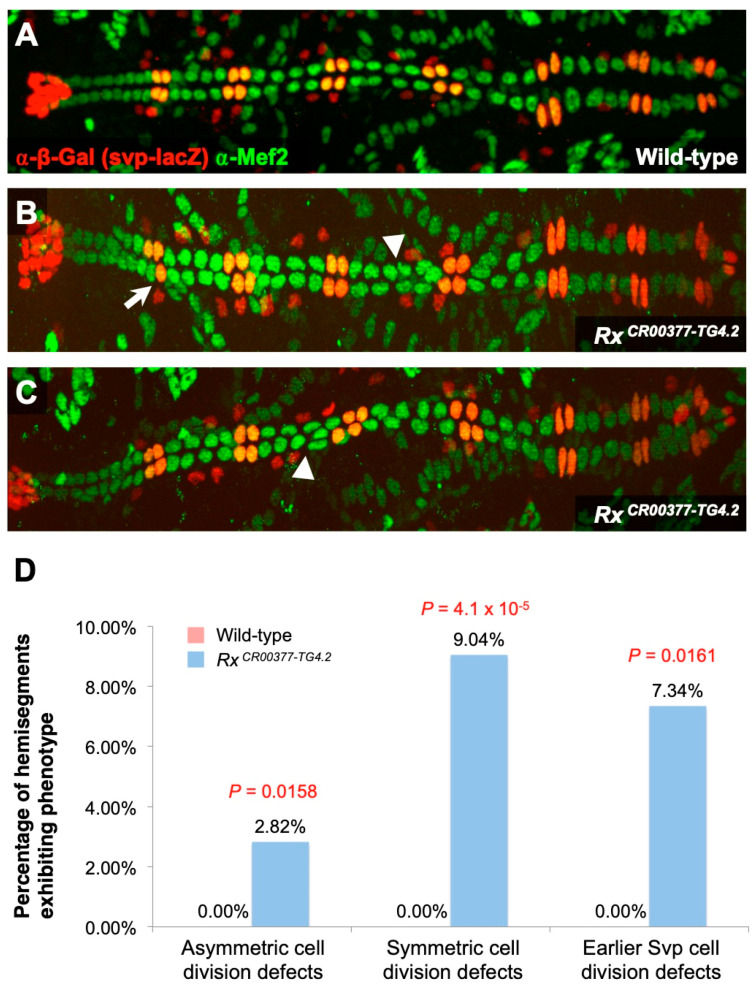
Cardiac progenitor cell division defects associated with an *Rx* hypomorphic mutation. (**A**) A heart from an otherwise wild-type embryo bearing one copy of the *svp-lacZ* enhancer trap showing hemisegments consisting of four Tin-CCs (green), two Svp-CCs (yellow), and two Svp-PCs (red). (**B**,**C**) Hearts from embryo homozygous for the *Rx ^CR00377-TG4.2^* hypomorphic mutation (and also carrying one copy of the *svp-lacZ* enhancer trap) exhibiting both cardiac progenitor symmetric cell division defects (arrowheads) and defects at an earlier round of cell division specifying the number of Svp progenitors (arrows). In these images (derived by flattening z-stacks), a few Svp-PCs are hidden underneath the CCs or appear quite faint in certain hemisegments, but all were clearly discernible in the individual planes of the z-stacks from which the images were obtained. (**D**) Percentage of hemisegments exhibiting each type of cardiac progenitor cell division defect in embryos that are wild-type (*n* = 196 hemisegments) or homozygous for the *Rx ^CR00377-TG4.2^* mutation (*n* = 177 hemisegments). The significance of each type of cell division defect in the *Rx* mutants compared with wild-type is shown.

Our investigation identified 21 *jumu*-activated genes mediating cardiac progenitor cell divisions. As proof of principle of the methodologies we intend to use in analyzing the functions of these *jumu*-regulated genes in bringing about cardiac progenitor cell division, we elected to examine the role of one of these genes in greater detail. The gene *Rx* was selected for this purpose both because it showed the greatest degree of relative reduction in expression in *jumu*-deficient mesodermal cells compared to wild-type among these 21 genes (Table 1), and because it was not present in any of the enriched annotation clusters of *jumu*-activated genes associated with cardiac progenitor cell division (i.e., Annotation Clusters 1, 2, 4, 5, 7, 8, and 11 in Section 2.2 and Supplementary Table S2D). As described earlier, we introduced the *svp-lacZ* enhancer trap into embryos that were otherwise wild-type as well as in embryos that were homozygous for the *Rx^CR00377-TG4.2^* mutant allele to determine the numbers of Tin-CCs, Svp-CCs, and Svp-PCs in every hemisegment and identify the types of cell division defects caused by the *Rx* mutant. Our examination revealed that embryos homozygous for the *Rx* mutant allele exhibited significant increases over wild-type for defects in all three categories of cardiac progenitor cell division—asymmetric cell division (*p* = 0.0158), symmetric cell division (*p* = 4.01 × 10^−5^), and cell division at the earlier stage (*p* = 0.0161)—showing that *Rx* mediates the same cardiac progenitor cell division processes that *jumu* does (Figure 4; Supplementary Table S4).

### 2.6. Synergistic Genetic Interactions Between Rx and Jumu

**Figure 5:**
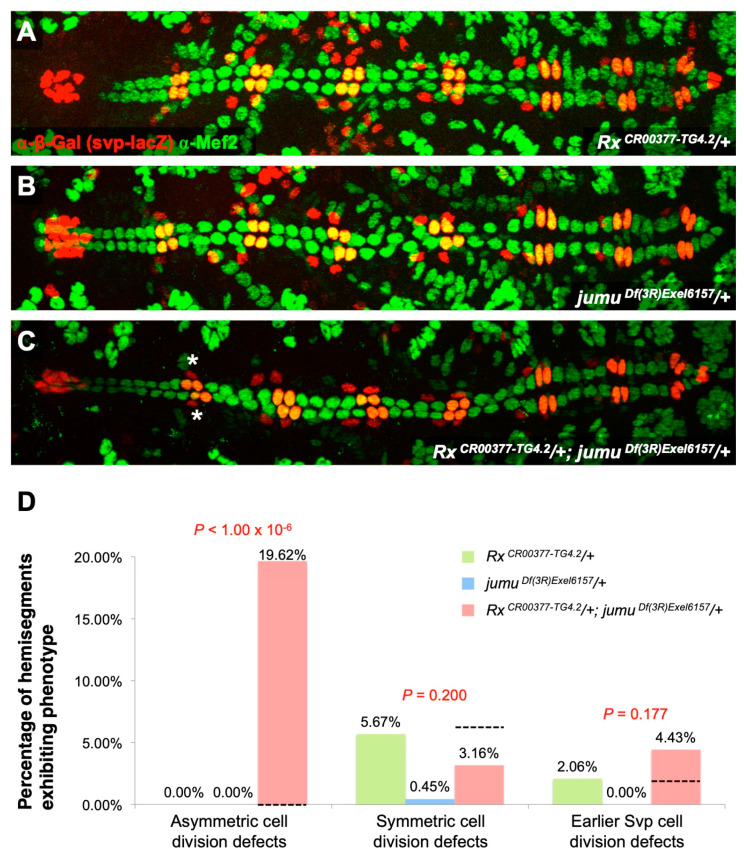
*Rx* exhibits synergistic genetic interactions with *jumu* in mediating asymmetric cardiac progenitor cell divisions. Representative hearts from embryos (**A**) heterozygous for the *Rx ^CR00377-TG4.2^* mutation, (**B**) heterozygous for the *jumu^Df(3R)Exel6157^* deficiency, and (**C**) doubly heterozygous for both the *Rx ^CR00377-TG4.2^* mutation and the *jumu^Df(3R)Exel6157^* deficiency. All of these embryos carry one copy of the *svp-lacZ* enhancer trap, thereby allowing the identification of Tin-CCs (green), Svp-CCs (yellow), and Svp-PCs (red). In these images (derived by flattening z-stacks), a few Svp-PCs are hidden underneath the CCs or appear quite faint in certain hemisegments, but all were clearly discernible in the individual planes of the z-stacks from which the images were obtained. Asymmetric cell division defects are denoted by asterisks. (**D**) The percentage of hemisegments exhibiting each type of cardiac progenitor cell division defect in embryos that are heterozygous for the *Rx ^CR00377-TG4.2^* mutation (*n* = 194 hemisegments), heterozygous for the *jumu^Df(3R)Exel6157^* deficiency (*n* = 224 hemisegments), or doubly heterozygous for both the *Rx ^CR00377-TG4.2^* mutation and the *jumu^Df(3R)Exel6157^* deficiency (*n* = 158 hemisegments). The black dashed line indicates the expected results in the double heterozygotes if the phenotypes were purely additive.

The observation that *jumu* activates *Rx* expression and that *Rx* mediates the same categories of cardiac progenitor cell division as *jumu* suggests that *jumu* may be acting through *Rx* to bring about these cell divisions: i.e., jumu and *Rx* could be functioning through the same genetic pathway. If this hypothesis is correct, then we may expect to detect synergistic, i.e., more than merely additive, genetic interactions between mutations or deficiencies of *jumu* and *Rx*. To assess this possibility, we quantitated and compared the cardiac progenitor cell division defect phenotypes of single heterozygotes of the *Rx* mutation and single heterozygotes of the *jumu* null deficiency with those of embryos that were doubly heterozygous for both the *Rx* mutation and the *jumu* deficiency (Figure 5).

Double heterozygotes for *Rx* and *jumu* exhibited only asymmetric cell division defects at frequencies that were significantly more severe (*p* < 1 × 10^−6^) than the additive sum of both the *Rx* single heterozygotes and the *jumu* single heterozygotes (Figure 5; Supplementary Table S5). These results indicate that *jumu* and *Rx* function through the same pathway mediate asymmetric cardiac progenitor cell divisions, providing strong support for the hypothesis that *jumu* utilizes *Rx* to bring about this cardiogenic process.

### 2.7. Chromatin Immunoprecipitation Data Suggests That 13 of the Jumu-Activated Genes Mediating Cardiac Progenitor Cell Divisions May Be Direct Transcriptional Targets of the Jumu TF

Not all *jumu*-regulated genes will necessarily be direct transcriptional targets of the Jumu TF, i.e., have associated *cis*-regulatory modules (CRMs) to which the Jumu TF binds to regulate transcription of the gene. Some may well be indirect targets, being regulated by the binding of other TFs, which were, in turn, regulated by the direct binding of Jumu to their CRMs. Thus, a particularly germane question is how many of these 21 *jumu*-activated genes mediating cardiac progenitor cell division are likely to be direct transcriptional targets of the Jumu TF.

To address this question, we took advantage of the modERN resource, which compiles genome-wide, chromatin immunoprecipitation sequencing (ChIP-seq)-based binding profiles for numerous TFs [63]. Previous analyses showed that the CRMs driving cardiac genes comprise clustered binding sites of multiple TFs critical for heart development and are located in either introns or the intergenic regions adjacent to these genes [41,64,65,66,67,68,69,70,71,72,73,74]. Hence, *jumu*-activated genes that are direct transcriptional targets of the Jumu TF would be expected to be associated with CRMs that contain both Jumu binding sites and those of other TFs mediating heart development. We therefore used the modERN resource to scan the intronic and intergenic regions of these 21 *jumu*-activated cardiac progenitor cell division-mediating genes for the presence of embryonic stage ChIP-binding peaks for Jumu, the Myb oncogene-like (Myb) TF that works in concert with Jumu to mediate cardiac progenitor cell divisions [36,38], and the following TFs known to play critical roles in heart development and regulate cardiac enhancers: Tinman (Tin), Twist (Twi), Tailup (Tup), Mothers against dpp (Mad), Pointed (Pnt), Suppressor of Hairless (Su(H)), and Hand [36,64,65,69,73,75,76,77,78,79,80,81,82,83,84]. Of note, our previous work had already shown that Fox TF (Jumu) binding sites were enriched along with multiple combinations of subsets of Tin, Twi, Mad, and Pnt binding sites in the CRMs of cardiac genes [36,41].

We considered a gene likely to be a direct transcriptional target of the Jumu TF if it exhibited a ChIP peak for Jumu TF binding clustered with a peak for at least one of the aforementioned TFs. Using this criterion, we found that the following 13 genes were likely to be direct targets of Jumu: *neb*, *sti*, *tum*, *SMC2*, *BubR1*, *barr*, *cmet*, *mei-S332*, *Cenp-C*, *Cdk2*, *scra*, *Incenp*, and *glu* (Supplementary File S1). In contrast, we found that *Apc2*, *Rx*, *pav*, *thr*, *pim*, *αTub67C*, *Klp61F*, and *cid* failed to show any embryonic stage Jumu TF binding peak, suggesting that their regulation by *jumu* was achieved in a more indirect manner (Supplementary File S1).

### 2.8. The 21 Jumu-Activated Cardiac Progenitor Cell Division-Mediating Genes Constitute Multiple Interaction Networks

Since all 21 of the *jumu*-activated genes we examined mediate cardiac progenitor cell divisions, another intriguing question is whether they each act independently to bring about this process or whether subsets of these genes act collectively in a pathway or network. To address this question, we utilized the Molecular Interaction Search Tool (MIST) resource [85]. MIST is an integrated database of curated biological interaction data for the major model organisms *Drosophila melanogaster*, *Mus musculus*, *Rattus norvegicus*, *Caenorhabditis elegans*, *Saccharomyces cerevisiae*, *Schizosaccharomyces pombe*, *Danio rerio*, *Xenopus laevis* and *Xenopus tropicalis*, and humans, *Homo sapiens*. In addition to displaying the known interactions among a pool of genes in a particular species, by mapping data among all these organisms using the DRSC Integrative Ortholog Prediction Tool (DIOPT) [86], MIST is able to provide orthology-based inferred interactions or interologs.

**Figure 6:**
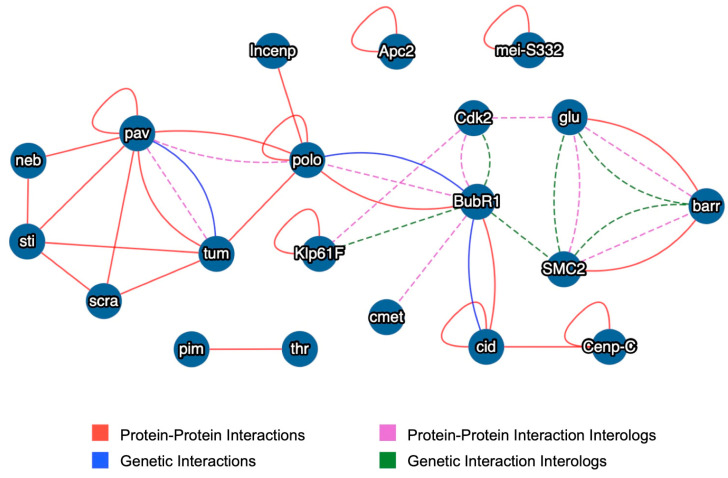
Inferred interaction networks derived by querying the MIST resource with a gene set comprising *polo* and the 21 *jumu*-activated cardiac progenitor cell division-mediating genes. Known protein–protein interactions and genetic interactions in *Drosophila* are illustrated as red and blue unbroken lines, respectively. Inferred protein–protein interaction interologs and genetic interaction interologs based on orthologous model systems are shown as purple and green dashed lines, respectively.

We therefore queried MIST with *polo* and the list of 21 *jumu*-activated cardiac progenitor cell division-mediating genes from this study for genetic and protein–protein interaction both in *Drosophila* and in other species using a stringent criterion that filtered out low ranking results. Parameters were set to only report direct pairwise interactions between these 22 *jumu*-activated genes or their orthologs. We intentionally omitted *jumu* from this pool, since all of these genes were already regulated by Jumu, and our goal was to identify which of them, if any, worked in concert to define specific *jumu*-regulated pathways mediating cardiac progenitor cell divisions. Our analysis revealed that 15 of these genes—*barr*, *BubR1*, *Cdk2*, *Cenp-C*, *cid*, *cmet*, *glu*, *Incenp*, *Klp61F*, *neb*, *pav*, *scra*, *SMC2*, *sti*, and *tum*—and/or their orthologs comprised a network incorporating *polo* while two other genes, *pim* and *thr*, constituted yet another network (Figure 6). Only four genes, *αTub67C*, *Apc2*, *mei-S332*, and *Rx*, failed to show such interactions with other genes in the pool. Since Jumu activates each of these 22 cardiac progenitor cell division-mediating genes, and 18 of these 22 genes comprise two inferred interaction networks, our data suggest that *jumu* is a master regulator of cardiac progenitor cell division networks.

## 3. Discussion

Our previous work had identified the conserved roles of two *Drosophila* Fox genes, *jumu* and *CHES-1-like*, in cardiogenesis [35,36,37,38]. Given the critical importance of Fox TFs in mammalian and human heart development and disease [3,4,5,6,7,8,9,10,11,12,13,14,15,16,17,18,19,20,21,22,23,24,25,26], our limited knowledge of the genes and molecular pathways through which Fox TFs mediate cardiogenesis, and the conservation of developmental processes between mammals and *Drosophila*, our goal in this study was to identify the downstream targets of one of these *Drosophila* Fox TFs, Jumu, and determine the cardiogenic processes they mediate, in the hope that it will shed light on related mechanisms in mammals.

Since *jumu* is also involved in multiple other non-cardiogenic processes such as neural fate specification, eye development, wing morphogenesis, immune response, and wound healing [42,43,44,45,46,47,48,49,50], we were concerned that attempting to identify cardiogenic *jumu*-regulated genes simply by comparing expression profiles obtained from whole-embryo RNA of wild-type and *jumu*-deficient embryos might prove difficult. A non-cardiogenic gene that exhibits no *jumu*-regulated expression change in the tissue of interest (mesodermal cells) would still have been identified as a (false positive) candidate if it showed a significant change in expression in some other non-relevant tissue. Alternatively, a true target gene could potentially be upregulated in one tissue while being downregulated in another, thereby exhibiting no significant total change in expression in the entire embryo, or have its *jumu*-regulated change in expression in the tissue of interest be swamped by the “noise” in the rest of the embryo. To guard against these possibilities, we chose to compare expression profiles from RNA extracted from purified mesodermal cells, the precursor of the embryonic heart. And, as detailed earlier in Section 2.4, our subsequent comparison with RT-qPCR analysis of whole embryo RNA indicated that obtaining and comparing expression profiles from purified relevant tissue subsets are more sensitive at identifying cardiogenic *jumu*-regulated downstream genes. We emphasize further that our approach, using the binary *GAL4-UAS* technique [87] with *UAS-2EGFP* to purify and obtain transcription expression only from cells of the appropriate tissue and genotypes, not only increases sensitivity and reduces potentially confounding effects, but is eminently generalizable and flexible. While we recombined the *jumu^Df(3R)Exel6157^* deficiency onto chromosomes containing either the *twist-GAL4* driver or the *UAS-2EGFP* transgene to purify and obtain expression profiles from mesodermal cells lacking *jumu* function in this study, we note that another feasible approach would be to use an appropriate *GAL4* driver to drive both a *UAS-2EGFP* transgene and a UAS-RNA interference (*UAS-RNAi*) construct for a particular gene to isolate cells from the relevant tissue where that gene had been knocked down. Nor does our approach for identifying downstream targets via comparison of transcription expression profiles from a targeted tissue of interest have to be limited solely to loss of function or knockdown of the specific gene being studied. The *GAL4* driver could also be used to drive a UAS-complementary DNA (*UAS-cDNA*) construct for a specific gene along with *UAS-2EGFP* to secure expression profiles from a targeted tissue where the gene of interest was overexpressed. Consequently, we believe that our approach to identify downstream targets of TFs with a high degree of sensitivity can be easily extended both to other developmental processes besides cardiogenesis and in systems other than *Drosophila*.

The enormous number of *jumu*-regulated genes that we identified via expression profiling in the embryonic mesoderm led us to examine these downstream genes for overrepresentation of functional annotation terms that could shed light on possible cardiogenic processes. As described earlier in Section 2.2, multiple enriched annotation clusters of *jumu*-activated genes identified in our analysis suggested that a large subset of these *jumu*-regulated genes could be mediating cardiac progenitor cell divisions. This hypothesis proved to be correct, since our subsequent phenotypic examination of 21 *jumu*-activated genes showed that each and every one of them was required for proper cardiac progenitor cell divisions.

Our results here highlight several important issues. First, not all of the *jumu*-activated genes we assessed phenotypically and found to mediate cardiac progenitor cell divisions (Figure 2 and Figure 3), such as *BubR1*, *pim*, and *Rx*, were identified in our functional annotation enrichment analysis (Supplementary Table S2D,E). This is a consequence of the functional annotation terms and ontology features listed in DAVID and used for our enrichment analysis, which was derived only from known genes that have undergone the appropriate functional characterization. It is thus very likely that many other uncharacterized or only partially characterized genes identified as being activated by *jumu* in our expression profile comparison are also required for cardiac progenitor cell division. This emphasizes the need to phenotypically assess the roles of these uncharacterized *jumu*-regulated genes to identify previously unknown players mediating this process—a process we are presently pursuing in our laboratory. Of note, many of the *jumu*-activated genes we found mediating cardiac progenitor cell divisions bring about cell division in other tissues as well. Thus, it is likely that in identifying some of the previously uncharacterized Jumu targets mediating cardiac progenitor cell divisions we might discover genes that have a more essential role in controlling cell division globally.

Second, while we focused on *jumu*-activated genes that were found to mediate cardiac progenitor cell divisions in this study, our functional annotation enrichment analysis revealed an overrepresentation of ECM protein-encoding genes among the *jumu*-repressed mesodermal genes. Loss or reduced function of these ECM genes in *Drosophila* has been associated with detachment of PCs from the heart tube, improperly aligned CCs, smaller lumens, and increased longevity resulting from the mitigation of the age-related decline in fractional shortening [88,89,90,91,92]. It will thus be interesting to explore what the effects of overexpression of these ECM genes, the consequence of a lack of *jumu* function, will be in the heart.

Third, there were many enriched annotation clusters for both *jumu*-activated and *jumu*-repressed mesodermal genes that featured terms, e.g., membranes and ion channels, that we could not necessarily be ascribed to one or more particular cardiogenic processes a priori. But that may simply reflect our incomplete knowledge and understanding of cardiogenic processes. Functional analysis of the genes comprising these annotation cluster that are not presently known to describe or define cardiogenic processes may shed yet more light on heart development.

Finally, the individual enriched annotation clusters themselves reflect only what is currently known. Many *jumu*-regulated genes might be involved in cardiogenic processes for which no enriched annotation cluster exists, either because that process has not yet been examined, or because too few of the genes mediating that process are *jumu*-regulated to constitute an overrepresented cluster. It is thus imperative to functionally analyze the *jumu*-regulated genes in an unbiased manner, regardless of whether a given gene is present in an enriched annotation cluster or not.

Nevertheless, the methodology we used in this study identified 21 *jumu*-activated genes that mediate cardiac progenitor cell divisions. We note that additional work needs to be carried out to characterize these genes further: determining which of the three distinct categories of cardiac progenitor cell division—symmetric, asymmetric, and/or earlier—each of these Jumu target genes mediate and assessing whether *jumu* also functions synergistically with the target gene through that particular cell division pathway. In this study, using *Rx* as an example, and in a parallel project where we carried out a detailed analysis of yet another of these Jumu targets, *neb* [38], we illustrate how to perform this functional characterization of the *jumu*-activated genes mediating cardiac progenitor cell divisions. It is also quite likely that several of these genes will work collectively through the same cardiac progenitor cell division pathways, a hypothesis supported by the interaction networks constituting many of these Jumu targets inferred by the MIST resource in this study. Also consistent with this hypothesis are previous reports noting that mutations in *αTub67C*, *Apc2*, *barr*, *BubR1*, *IncenP*, *Klp61F*, *neb*, *pav*, and *thr* result in an aberrant spindle structure, position, or orientation [93,94,95,96,97,98,99,100,101,102,103,104]. Note, however, that the inferred networks were often based on interactions between orthologs of these genes in other species. Thus, once the initial characterization of all 21 of these genes is complete, genetic interaction, epistasis, and rescue assays should be used to determine the composition and topology of the actual pathways or networks that these genes constitute in *Drosophila* to bring about proper *jumu*-regulated cardiac progenitor cell divisions.

Using chromatin immunoprecipitation data, we have also suggested that 13 of the 21 *jumu*-activated cardiac progenitor cell division-mediating genes identified in this project are likely to be directly regulated by Jumu TF binding. However, confirming whether any of these 13 likely candidates were indeed direct transcriptional targets of the Jumu TF was beyond the scope of this initial project. For each of these candidate genes, this project would entail first creating and utilizing transgenic enhancer–reporter constructs to identify and define the CRM that drives its expression: enhancer–reporter constructs incorporating the correct CRM would drive reporter expression with the same pattern as the endogenous candidate gene. Once the CRM was identified, *cis-* (mutating the Jumu TF binding site in the enhancer–reporter construct) and *trans-* (examining the effect on the unmutated enhancer–reporter construct in *jumu*-deficient embryos) assays would be deployed: if the candidate gene was indeed a direct transcriptional target of Jumu, then the disruption of Jumu TF binding to the CRM in both assays should significantly alter reporter activity from the enhancer–reporter construct. Given our prior experience in identifying CRMs and using such *cis*- and *trans*- assays [35,36,41,82], we intend to assess which of these genes are direct targets of Jumu in the future as we functionally characterize each gene in greater detail.

Given our professed goal for undertaking this study, a particularly germane question is whether the *jumu*-regulated genes we identified in *Drosophila* and the pathways or networks they constitute will prove to be conserved and thus shed light on orthologous Fox TF-regulated cardiogenic processes in vertebrates, and, by extension, in humans. Since this study identified *jumu* as a master regulator of cardiac progenitor cell divisions in *Drosophila*, it was gratifying to note that at least two mammalian Fox genes, *Foxm1* and *Foxp1*, are also essential for proper cardiomyocyte proliferation [12,13,17,105,106]. Also consistent with potential conserved roles in cardiogenesis, the mammalian orthologs of at least 7 of these 21 *jumu*-activated cardiac progenitor cell division-mediating genes—*αTub67C* (*TUBA8*/*Tuba8* in humans and mice), *Cdk2* (*CDK14* in humans), *neb* (*KIF1C* and *KIF16B* in humans), *pav* (*KIF20A* in humans), *scra* (*Anln* in mice), *SMC2* (*SMC3* in mice), and *sti* (*CDC42BPA*, *CDC42BPB*, and *CDC42BPG* in humans and *Rock1* in mice)—are expressed at high levels in the mammalian heart [107,108,109,110,111,112,113,114,115,116,117,118]. In addition, mutations in the *Apc2* zebrafish ortholog *apc* result in heart looping defects reminiscent of those in *Foxa2* and *Foxo1* knockouts in mice [3,6,14,119], mutations in the *neb* human ortholog *KIF14* are linked with congenital heart defects associated with the Meckel-Gruber syndrome [120], the *pav* zebrafish ortholog *kif20a* is essential for heart development while mutations in the human *KIF20A* cause familial restrictive cardiomyopathy-6 [116,121], and mutations in the *SMC2* human ortholog *SMC3* are associated with high rates of congenital heart disease [122]. Finally, our querying of the MIST resource with the 21 *jumu*-activated cardiac progenitor cell division-mediating genes identified in this study indicated that at least 15 of them constituted inferred interaction networks (Figure 6). These interaction networks were inferred not merely on known genetic or protein–protein interaction solely between the *Drosophila* genes, but also on known interactions between the orthologs of these genes in other model systems, suggesting a considerable degree of conservation. Collectively, these data indicate that the genes acting downstream of the *Drosophila* Fox gene *jumu*, and the pathways and networks they comprise, are indeed conserved between multiple species and will prove particularly useful in understanding Fox TF-mediated cardiogenic processes in mammals and humans.

## 4. Materials and Methods

### 4.1. Drosophila Strains

The following deficiencies, mutant alleles, and transgenes were used: *jumu^Df(3R)Exel6157^* (also known as *Df(3R)Exel6157*; FlyBase ID: FBab0038212) [37], *twi-GAL4* (also known as *Scer\GAL4^twi.PG^*; FlyBase ID: FBal0040491) [52], *UAS-2EGFP* (also known as *Avic\GFP* ^*2x.EGFP.UAS*^; FlyBase ID: FBal0128321) [53], *αTub67C* ^*1*^ (FlyBase ID: FBal0000008) [123], *Apc2^d40^* (FlyBase ID: FBal0137665) [124], *barr ^L305^* (FlyBase ID: FBal0057771) [125], *BubR1^k03113^* (FlyBase ID: FBal0064564) [126], *Cdk2^2^* (FlyBase ID: FBal0117572) [127], *Cenp-C* ^*SH157*^ (FlyBase ID: FBal0294004) [128], *cid ^T11-2^* (FlyBase ID: FBal0221673) [129], *cmet* ^*04431*^ (FlyBase ID: FBal0008060) [130], *glu ^k08819^* (FlyBase ID: FBal0043098) [131], Incenp ^QA26^ (FlyBase ID: FBal0012084) [132], *Klp61F ^URC-1^* (FlyBase ID: FBal0032985) [133], *mei-S332^3^* (FlyBase ID: FBal0032116) [134], *neb ^k05702^* (FlyBase ID: FBal0043429) [135], *pav ^963^* (FlyBase ID: FBal0178488) [136], *pim^249^* (FlyBase ID: FBal0337959) [137], *Rx^CR00377-TG4.2^* (FlyBase ID: FBal0340980), *scra ^03427^* (FlyBase ID: FBal0008026) [138], *SMC2 ^jsl2^* (FlyBase ID: FBal0191670) [139], *sti^12C001^* (FlyBase ID: FBal0302670) [140], *thr ^2^* (FlyBase ID: FBal0016787) [104], *tum ^DH15^* (FlyBase ID: FBal0176032) [141], and *svp-lacZ* (also known as *svp^3^*; FlyBase ID: FBal0016610 [61].

### 4.2. Genetic Crosses, Cell Suspension Preparation, and Flow Cytometry Used to Isolate Purified Populations of Wild-Type and Jumu-Deficient Mesodermal Cells from Drosophila Embryos

Two distinct sets of crosses were used to purify wild-type mesodermal cells and mesodermal cells lacking *jumu* function:

- *twi-GAL4 UAS-2EGFP* × +/+
  All GFP-positive cells were wild-type mesodermal cells of the genotype *twi-Gal4 UAS-2EGFP/+*.
- *twi-GAL4 jumu^Df(3R)Exel6157^/TM3 × UAS-2EGFP jumu^Df(3R)Exel6157^/TM3*
  The deficiency *jumu^Df(3R)Exel6157^* completely deletes the *jumu* gene; all GFP-positive cells were mesodermal cells of the genotype *twi-GAL4 jumu^Df(3R)Exel6157^/UAS-2EGFP jumu^Df(3R)Exel6157^* and lacked *jumu* function.

Four biological replicates were performed for each cross. Embryos from each of these crosses were collected and aged at 25 °C to 6–8 h after egg deposition, dechorionated by incubation for 5 min in 50% bleach, and rinsed successively in 0.01% Triton X-100 and distilled water. The embryos were then gently dissociated in a loose-fitting Dounce homogenizer (VWR: catalogue # 62400-620) in 7 mL of Schneider’s Drosophila medium with the suspension being kept continuously on ice. The embryo suspensions were next centrifuged at 40XG for 5 min to pellet debris. The supernatant was then centrifuged at 380XG for 10 min to pellet the cells, which were resuspended in Schneider’s Drosophila medium and sieved through a 40 µm nylon mesh to ensure a single cell suspension.

Using cells from wild-type embryos lacking both *twi-GAL4* and *UAS-2EGFP* for comparison as GFP-negative control cells, the GFP-expressing mesodermal cell populations described above were isolated at >90% purity from the single-cell suspensions via florescence-activated cell sorting (FACS). FACS was performed using standard protocols, with the following modifications: the running buffer used for *Drosophila* cells was Seecof saline (6 mM Na_2_HPO_4_, 3.67 mM KH_2_PO_4_, 106 mM NaCl, 26.8 mM KCl, 6.4 mM MgCl_2_, 2.25 mM CaCl_2_, pH 6.8); the machine was cooled to 4 °C during sorting; collection was into 1.5 mL aliquots of RNAlater (Ambion/ThermoFisher, Waltham, MA, USA) on ice. The sorted cell suspensions were then diluted with Schneider’s Drosophila medium such that the RNAlater was no more than 20% of the total volume and centrifuged at 5000XG for 15 min. The resulting cell pellets were resuspended in Trizol (Invitrogen/ThermoFisher, Waltham, MA, USA) and total RNA was extracted according to the manufacturer’s protocols.

### 4.3. RNA Sequencing and Data Analysis

RNA libraries for RNA-seq were prepared using the Illumina TruSeq stranded total RNA library preparation kit following the manufacturer’s protocols. RNA-seq was performed on an Illumina HiSeq 2500.

RNA-seq transcriptome profiling was carried out for 8 samples: 4 biological replicates each of the purified wild-type and *jumu* loss-of-function mesodermal cells. On average, 28.2 million reads with 44.3% GC content were obtained. The RNA-seq reads were mapped to FlyBase using the dmel_r6.05_FB2015_02 annotation [142,143] by TopHat2 [144] with default parameters. The average mapping rate was about 92%. Differential expression analysis between *jumu* loss-of-function and wild-type mesodermal cells was performed using the Bioconductor tool edgeR [145]. Genes with low counts per million (the bottom 30% with log_2_CPM after edgeR normalization, highlighted in green in Supplementary Table S1) were excluded in our subsequent analysis.

### 4.4. Functional Annotation Enrichment Analysis

The DAVID knowledgebase [54,55] was queried independently for functional annotation clustering with each of two gene sets: the *jumu*-repressed genes (relaxed stringency; log_2_FoldChange > 0.5 and False Discovery Rate < 0.1; 1250 genes) and *jumu*-activated genes (relaxed stringency; log_2_FoldChange < −0.5 and False Discovery Rate < 0.1; 1246 genes). In both cases, all genes of the *Drosophila melanogaster* genome were used as the background.

### 4.5. Immunohistochemistry, Microscopy, and Cell Counting

Embryo fixation and fluorescent immunohistochemistry were performed as described previously [35,37]. The following primary antibodies were used: rabbit anti-Mef2 (1:1000 dilution, from the Developmental Studies Hybridoma Bank; DSHB Catalog no. rab Mef2, RRID:AB_2892602), mouse anti-β-galactosidase (1:500 dilution, Promega Catalog no. Z3783, RRID:AB_430878), chicken anti-β-galactosidase (1:500 dilution, Abcam Catalog no. ab9631, RRID:AB_307210), and mouse anti-Svp (1:5 dilution, monoclonal 5B11 from the Developmental Studies Hybridoma Bank; DSHB Catalog no. Seven-up 5B11, RRID:AB_2618080). Fluorescence microscopy was performed on a Zeiss AxioImager with Apotome. Z-stacks of entire stage 16 embryonic hearts were scanned with a 40X objective and 0.31 µm steps, and all planes for each z-stack were examined to count cells and determine cell division defects. Cell counting and assessment of cell division defects were performed blind (i.e., the individual evaluating cardiac progenitor cell division defects did not know the genotypes of the embryos being assessed) to avoid any potential bias.

### 4.6. Statistical Analysis of Cell Division Defects

Comparison of cell division error rates between genotypes was performed using regression models with the response variable being the proportion of hemisegmental errors for each embryo. Due to violation of regression assumptions, e.g., non-normality and heteroscedasticity, permutation (randomization) tests were conducted using R, version 4.2.2 to obtain reliable *p*-values [146].

For comparing rates between two genotypes, for example, *αTub67C* ^*1*^ and wild-type, the following general linear model was used:

$$Y_{j}=\beta_{0}+\beta_{1}I_{j}+\varepsilon_{j},$$

where $Y_{j}$ is the proportion of hemisegmental errors for embryo *j* and indicator variable $I_{j}$ is 1 if embryo *j* has phenotype *αTub67C ^1^* and 0 otherwise. To obtain a permutation *p*-value for testing $H_{0}:\beta_{1}=0$, the estimate of $\beta_{1}$ for the actual data is compared with the estimates obtained when the genotypes of the embryos are permuted, i.e., the phenotype labels are randomly shuffled among the embryos in the sample. The permutation *p*-value is then p=(n+1)/(N+1) where n is the number of permutation estimates for which $\beta_{1}$ equals or exceeds the estimate for the actual data and N is the number of permutations [147]. In order to obtain highly reproducible *p*-values, $N=10^{6}$ permutations were used for all permutation tests.

For determining if cell division error rates are non-additively related to two gene mutations, for example, to detect synergistic interaction between *Rx* and *jumu*, a general linear model allowing for interaction was used:

$$Y_{j}=\beta_{1}I_{p,j}+\beta_{2}I_{q,j}+\beta_{3}I_{p,j}I_{q,j}+\varepsilon_{j},$$

where $I_{p,j}$ is 1 if the *j*th embryo is heterozygous for the *Rx^CR00377-TG4.^*^2^ mutation and 0 otherwise and $I_{q,j}=1$ only if it is heterozygous for the *jumu^Df(3R)Exel6157^* deficiency. Since synergism is present only if $\beta_{3}\neq 0$, to detect it, $H_{0}:\beta_{3}=0$ was tested using permutation. Since this is a multiple regression model, a somewhat more sophisticated permutation procedure, the Smith procedure (orthogonalization), was employed [148].

### 4.7. Reverse Transcription Quantitative Real-Time PCR (RT-qPCR) Assays

Wild-type embryos and embryos that were homozygous for the *jumu^Df(3R)Exel6157^* deficiency were collected and aged at 25 °C to 6–8 h after egg deposition. Wild-type embryos were obtained simply by crossing wild-type parents, while the *jumu^Df(3R)Exel6157^* homozygotes were selected as GFP-negative embryos using a fluorescence microscope from the progeny of *jumu^Df(3R)Exel6157^ /TM3*, *twi-GAL4 UAS-2EGFP* parents. Approximately 70–80 embryos were pooled and used for each replicate. Embryos were dechorionated by immersion for 5 min in 50% bleach, and rinsed thoroughly in distilled water.

Total RNA was isolated immediately after the dechorionation and rinse step, cDNA was prepared, and RT-qPCR was performed in technical triplicates as described previously [38] using the following primer pair sets from the FlyPrimerBank database [149] for the 21 *jumu*-activated cardiac progenitor cell division-mediating genes: *αTub67C*-PP5028, *Apc2*-PP16425, *barr*-PP13879, *BubR1*-PP28751, *Cdk2*-PA60130, *Cenp-C*-PA60347, *cid*-PP25432, *cmet*-PP3809, *glu*-PP14780, *Incenp*-PP8912, *Klp61F*-PP19242, *mei-S332*-PP15231, *neb*-PP15139, *pav*-PP35876, *pim*-PP10356, *Rx*-PP15393, *scra*-PP35881, *SMC2*-PP16142, *sti*-PP16838, *thr*-PP18025, and *tum*-PP22571. One-tailed, two-sample, unequal variance, heteroscedastic t-tests were used for statistical analysis. Relative gene expression was calculated using the 2^−ΔΔCT^ method [150].

### 4.8. Mapping of Chromatin Immunoprecipitation Data of Cardiogenic Transcription Factors

Chromatin immunoprecipitation (ChIP) data from the modERN database [63] were loaded to the UCSC genome browser track hub [151]. The NCBI Refseq data for the *Drosophila melanogaster* genome (BDGP Release 6 + ISO1 MT/dm6) were used to obtain the gene boundaries for the 21 *jumu*-activated cardiac progenitor cell division-mediating genes and the intergenic regions bounded by the adjacent genes immediately 5′ and 3′ to them. ChIP binding site peaks during embryonic stages for the cardiogenic TFs Jumu, Myb, Tin, Tup, Twi, Su(H), Pnt, Mad, and Hand were then plotted in each of these 21 genomic intervals.

### 4.9. Inference of Interaction Networks Using MIST Resource

The MIST resource [85] was queried with a gene set comprising *polo* and the 21 *jumu*-activated cardiac progenitor cell division-mediating genes using the following parameters:

- Species/model organism of interest: *Drosophila* (*D. melanogaster*).
- Search type: Protein list (find interactors within input).
- Networks to search: Protein–protein interactions; Interologs: protein–protein interaction from other species; Genetic interactions; and Interologs: genetic interactions from other species (with low-rank results filtered out for all four searched networks).

## Figures and Tables

**Table 1:** *jumu*-activated genes selected for phenotypic analysis.

Gene	log_2_FoldChange ^1^	*p*-Value ^2^	FDR ^2^
*αTub67C*	−0.952490176	0.000867	0.00578
*Apc2*	−0.639416425	3.91 × 10^−10^	8.498 × 10^−9^
*barr*	−0.869589586	1.83 × 10^−27^	1.24 × 10^−25^
*BubR1*	−1.07030746	5.28 × 10^−27^	3.47 × 10^−25^
*Cdk2*	−0.858035426	4.32 × 10^−11^	1.03 × 10^−9^
*Cenp-C*	−1.051992964	7.11 × 10^−28^	4.84 × 10^−26^
*cid*	−0.55623802	2.40 × 10^−5^	0.000242
*cmet*	−0.692319454	1.33 × 10^−17^	5.41 × 10^−16^
*glu*	−1.110534588	1.31 × 10^−37^	1.28 × 10^−35^
*IncenP*	−0.922209804	1.90 × 10^−21^	9.85 × 10^−20^
*Klp61F*	−0.554131891	8.16 × 10^−10^	1.71 × 10^−8^
*mei-S332*	−0.883435396	3.81 × 10^−18^	1.61 × 10^−16^
*neb*	−0.973540901	5.11 × 10^−23^	2.87 × 10^−21^
*pav*	−0.968485941	5.44 × 10^−36^	5.05 × 10^−34^
*pim*	−1.289603484	8.26 × 10^−22^	4.35 × 10^−20^
*Rx*	−3.294594189	6.71 × 10^−92^	2.39 × 10^−89^
*scra*	−1.042234664	4.60 × 10^−17^	1.82 × 10^−15^
*SMC2*	−1.095214765	2.59 × 10^−22^	1.41 × 10^−20^
*sti*	−1.291936939	2.90 × 10^−60^	5.52 × 10^−58^
*thr*	−0.679554768	2.06 × 10^−10^	4.63 × 10^−9^
*tum*	−1.221556643	1.20 × 10^−55^	1.94 × 10^−53^

^1^ The log_2_FoldChange column displays the relative reduction in the expression level of each gene in *jumu*-deficient mesodermal cells compared to wild-type cells. ^2^ The *p*-value and FDR (False Discovery Rate) columns exhibit the significance of these changes in expression levels.

## Data Availability

The original contributions presented in the study are included in the article and Supplementary Materials. The RNA-seq data are available from the Gene Expression Omnibus with the accession number GSE275809.

## Supplementary Materials

The following supporting information can be downloaded at: https://www.mdpi.com/article/10.3390/ijms252312933/s1.
