# Supplementary figures and images for: Genome-Wide Expression Profiling and Phenotypic Analysis of Downstream Targets Identify the Fox Transcription Factor Jumeau as a Master Regulator of Cardiac Progenitor Cell Division
Source: Int J Mol Sci. 2024 Dec 1;25(23):12933. doi: 10.3390/ijms252312933 (PMC11641245; doi:10.3390/ijms252312933)

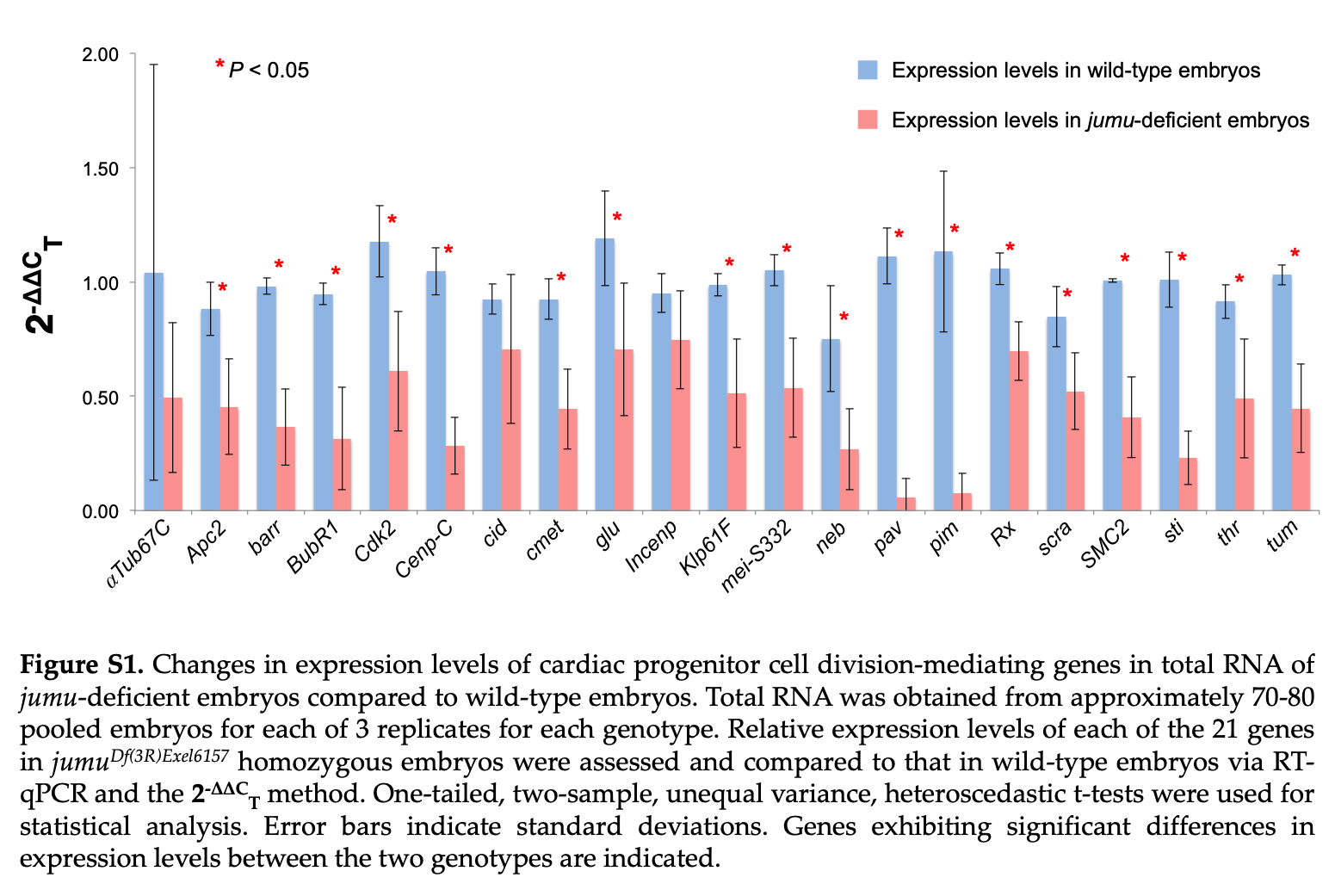

Supplement: Supplementary file 1 [file ijms-25-12933-s001.zip › FigureS1.tiff]
